# Decreased CD8^+^ Lymphocytic Infiltration in Multifocal and Multicentric Glioblastomas

**DOI:** 10.3389/fonc.2021.748277

**Published:** 2021-09-27

**Authors:** Run Wang, Yifu Song, Tianhao Hu, Xiaoliang Wang, Yang Jiang, Di Zhang, Juanhan Yu, Sheng Han, Liang Kan

**Affiliations:** ^1^ Department of Neurosurgery, The First Hospital of China Medical University, Shenyang, China; ^2^ Department of Neurosurgery, Huazhong University of Science and Technology Union Shenzhen Hospital (Nanshan Hospital), Shenzhen, China; ^3^ Department of Neurosurgery, Shanghai First People’s Hospital of Shanghai Jiao Tong University School of Medicine, Shanghai, China; ^4^ Department of Pathology, China Medical University, Shenyang, China; ^5^ Department of Geriatrics, Shengjing Hospital of China Medical University, Shenyang, China

**Keywords:** Multifocal glioma, CD8, tumor-infiltrating lymphocytes (TILs), chemotherapy, prognosis

## Abstract

**Purpose:**

Multifocal and multicentric glioblastomas (mGBMs) are associated with a poorer prognosis compared to unifocal glioblastoma (uGBM). The presence of CD8^+^ tumor-infiltrating lymphocytes (TILs) is predictive of clinical outcomes in human malignancies. Here, we examined the CD8^+^ lymphocytic infiltration in mGBMs.

**Methods:**

The clinical data of 57 consecutive IDH wildtype primary mGBM patients with histopathological diagnoses were retrospectively reviewed. CD8^+^ TILs were quantitatively evaluated by immunohistochemical staining. The survival function of CD8^+^ TILs was assessed by Kaplan–Meier analysis and Cox proportional hazard models.

**Results:**

No significant difference in the concentration of CD8^+^ TILs was observed among foci from the same patient (P>0.150). The presence of CD8^+^ TILs was similar between multifocal and multicentric GBMs (P=0.885). The concentration of CD8^+^ TILs was significantly lower in mGBMs than in uGBMs (P=0.002). In mGBM patients, the CD8^+^ TIL level was associated with preoperative KPS (P=0.018). The median overall survival (OS) of the 57 mGBMs was 9 months. A low CD8^+^ TIL level (multivariate HR 4.404, 95% CI 1.954-9.926, P=0.0004) was an independent predictor of poor OS, while postoperative temozolomide chemotherapy (multivariate HR 6.076, 95% CI 2.330-15.842, P=0.0002) was independently associated with prolonged OS in mGBMs.

**Conclusions:**

Decreased CD8^+^ TIL levels potentially correlate with unfavorable clinical outcome in mGBMs, suggesting an influence of the local immuno-microenvironment on the progression of mGBMs.

## Introduction

Glioblastoma (GBM) is the most common primary malignant tumor in the central nervous system (CNS) and exhibits a poor prognosis (1). GBM is therapeutically intractable and refractory to currently available multimodal treatments including surgery, radiotherapy, and chemotherapy (2). Most newly diagnosed GBMs present as a unifocal GBM (uGBM), whereas GBMs with multiple lesions are relatively rare, as the latter account for only 0.5%–20% of all GBMs based on histopathological or radiological diagnosis (3–9). Multiple GBMs are categorized into multifocal and multicentric GBMs (mGBMs). The foci of multifocal GBMs are close to each other, suggesting a physical connection; in contrast, the foci of multicentric are located in different lobes or hemispheres without any obvious dissemination route (10, 11). Nevertheless, prior publications have shown no significant utility in the distinction between multifocal and multicentric GBMs (5, 7, 10–13). Therefore, per the convention of previous studies (7, 10, 11), we use the term “mGBM” in the present study to represent our cases with multiple GBM lesions within a single patient. According to previous reports, mGBMs are associated with even worse clinical outcome and poorer survival times compared to uGBMs (7, 10, 14, 15). However, the pathogenic mechanisms and clinical characteristics of mGBMs are still largely unclear, and standards of care for mGBMs are not well defined. Therefore, new treatment strategies, including immunotherapies, need to be developed for mGBMs.

The tumor microenvironment (TME) is a critical regulator of pathogenesis and therapeutic responses in GBMs (16). CD8^+^ tumor-infiltrating lymphocytes (TILs) have been demonstrated to be important components of the TME and predict improved survival in several human malignancies, including breast cancer (17), ovarian cancer (18), and colorectal cancer (19). CD8^+^ TILs have also been detected in the TME of GBMs; however, the prognostic effect of CD8^+^ TILs in GBMs is still controversial (20–23). Moreover, the role of CD8^+^ TILs in mGBMs has not yet been elucidated. Thus, in the present study, we investigated the infiltration of CD8^+^ lymphocytes in a series of 57 newly diagnosed mGBMs and assessed the effects of CD8^+^ TILs and other clinical parameters on the prognosis of mGBM patients. Taken together, our findings might shed light on immuno-microenvironmental mechanisms of mGBM biology.

## Patients and Methods

### Study Patients

This study was approved by the institutional review board of the First Hospital of China Medical University (AF-SOP-07-1.1-01). All methods below were performed in accordance with the relevant guidelines and regulations. From June 2015 to May 2019, 492 patients were newly diagnosed with GBMs at the First Hospital of China Medical University. Among them, 57 consecutive patients (11.6%) had histopathological diagnoses of primary mGBMs and were included in the present study. All the examined foci from the 57 mGBMs were WHO grade IV and IDH wildtype, as determined by next-generation sequencing (Genetron, Shanghai, China). Methylation of the MGMT promoter was detected by a methylation-specific PCR-Fluorescent-Probe Method, as previously described (21). The histopathological diagnoses were reported by the Pathology Department of China Medical University and were further confirmed by two neuropathologists. The clinical data, radiological examinations, and follow-up information were retrospectively reviewed and analyzed. No patients had systemic diseases, other cancers, or other CNS tumors.

Tumor resection was determined by intraoperative observation and postoperative magnetic resonance imaging (MRI) and was defined as gross total resection (GTR, removal of all foci), partial resection (removal of one or more foci) and stereotactic biopsy. Tumor size was calculated as the sum size of all foci, based on preoperative enhanced MRI using the following formula: Σ (anteroposterior diameter × transverse diameter × axial diameter) × π/6. After the histopathological diagnosis was made, adjuvant therapies were determined by multidisciplinary discussion within a brain-tumor team that included neuroradiologists, neuropathologists, radiation oncologists, neurooncologists, and neurosurgeons. The final treatment plan also took the personal decision of each patient into consideration. Ultimately, 16 patients received 3D conformal radiotherapy (60 Gy in 2.0 Gy fractions) as previously described (15), 35 patients received temozolomide (TMZ) chemotherapy according to the Stupp protocol (2), and 15 patients did not receive radiotherapy or chemotherapy.

### Immunohistochemistry (IHC)

IHC staining and quantitative evaluation were performed as previously reported (21). Briefly, paraffin-embedded sections underwent deparaffinization with xylene, and rehydration and antigen retrieval was achieved *via* microwaving in 10 mmol/L of sodium citrate buffer (pH 6.0). After blocking endogenous peroxidase with 3% H_2_O_2_ in methanol and blocking non-specific binding with protein-blocking buffer, sections were incubated with primary antibody against CD8 (clone 144B, 1:50; Abcam, Cambridge, UK). Normal mouse serum was used as a negative control. Then, sections were incubated with a horseradish-peroxidase-labeled secondary antibody, colored with diaminobenzidine, and counterstained with hematoxylin. Results were observed and photographed under a light microscope connected to a computer (Olympus, Tokyo, Japan). Each section was assessed using at least five different high-power fields (HPF, 400×) with the most abundant CD8^+^ TILs. The concentration of CD8^+^ TILs was independently counted at least three times by two experienced neuropathologists (DZ and JY) blinded to the clinical backgrounds of the patients. To ensure reproducibility, the results were re-examined after a period of time. When a satisfactory intra-observer and inter-observer agreement was obtained, the average of CD8^+^ TIL counts per field for each patient was utilized for further statistical analysis as previously described (17, 21). Ki-67 IHC staining was routinely performed and reported by the Pathology Department of China Medical University.

### Statistical Analysis

The results are presented as the mean ± standard error of the mean (SEM). The *t* test, analysis of variance, and Mann-Whitney test were used to assess statistical significance. The Kaplan-Meier analysis and log-rank test were used to evaluate survival differences. Multivariate Cox analyses were used to calculate hazard ratios (HRs) of deaths according to different variables. All statistical analyses were performed using SPSS v25.0 (SPSS Inc., Chicago, IL) and GraphPad Prism 8 (GraphPad Software Inc., La Jolla, CA). Statistical significance was set at *P*<0.05 (two-tailed).

## Results

### Clinical Features

**Figure 1:**
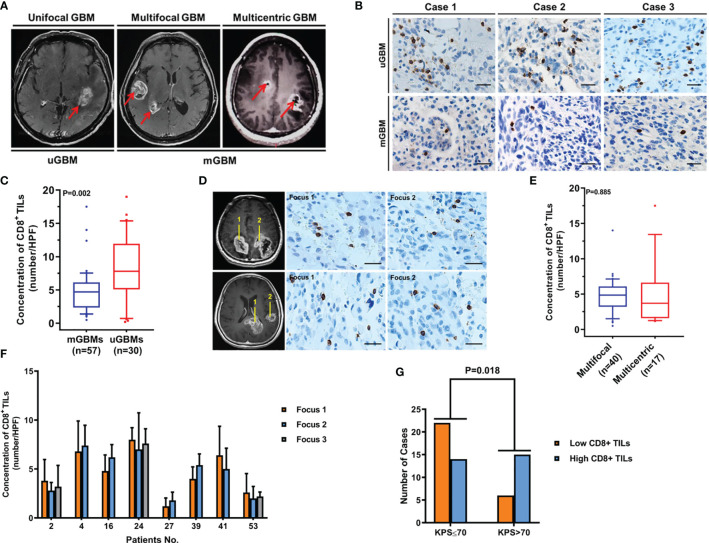
CD8^+^ TIL levels are decreased in mGBMs. **(A)** Typical MRIs of a unifocal GBM (uGBM), multifocal GBM, and multicentric GBM (mGBM). **(B)** Representative CD8^+^ TIL IHC images of uGBM and mGBM cases. Scale bar, 20 μm. **(C)** The concentration of CD8^+^ TILs was significantly lower in mGBMs than in uGBMs. **(D)** IHC staining of CD8^+^ TILs in different foci of multicentric and multifocal GBMs. Scale bar, 20 μm. **(E)** The presence of CD8^+^ TILs was similar between multifocal and multicentric GBMs. **(F)** In mGBMs, no significant difference in the concentration of CD8^+^ TILs was observed among foci from the same patient. **(G)** CD8^+^ TIL levels were significantly correlated with preoperative KPS.

As shown in **Table 1**, in this series of 57 patients, there were 45 male (78.9%) and 12 female (21.1%) patients, with an average age of 55.3 ± 8.9 years (39–68 years). Forty (70.2%) patients had multifocal GBMs, and seventeen (29.8%) patients had multicentric GBMs (**Figure 1A**). The mean tumor size was 21.8 ± 13.3 cm^3^ (0.2–82.3 cm^3^). Thirty-one (54.4%) patients received GTR, fifteen (26.3%) patients received partial resection, and eleven (19.3%) patients received biopsies. There were 23 (40.4%) cases with Ki-67 >50% and 34 (59.6%) cases with Ki-67 <50%. Furthermore, there were 29 (50.9%) patients with a methylated MGMT promoter. The median follow-up time was 9 months (ranging from 1 to 20 months), during which 35 patients died from GBMs, and no patient was lost to follow-up.

**Table 1:** Clinical feature of the 57 multifocal and multicentric glioblastomas.

Clinical feature	No.	%
Total	57	100
*Sex*		
Male	45	78.9
Female	12	21.1
*Age (years)*		
Mean ± SD	55.3 ± 8.9	
*Pre-operative KPS*		
≤80	36	63.2
>80	21	36.8
*Tumor size (cm^3^)*		
Mean ± SD	21.8 ± 13.3	
*Tumor resection*		
Gross total resection	31	54.4
Partial resection	15	26.3
Biopsy	11	19.3
*Ki67*		
<50%	34	59.6
>50%	23	40.4
*MGMT promoter*		
Methylated	29	50.9
Unmethylated	28	49.1
*Kind of mGBM*		
Multifocal	40	70.2
Multicentric	17	29.8
*Radio-chemotherapy*		
Radiotherapy	7	12.3
Chemotherapy	26	45.6
Both	9	15.8
Neither	15	26.3

KPS, Karnofsky performance status; MGMT, O(6)-methylguanine-DNA-methyltran –sferase; mGBM, multifocal and multicentric glioblastoma.

### CD8^+^ Lymphocytic Infiltration in mGBMs

To compare CD8^+^ lymphocytic infiltration in mGBMs with that in uGBMs, CD8^+^ TILs were detected in 57 mGBMs and 30 uGBMs. As shown in **Table 2**, the mGBMs and uGBMs were matched in terms of sex, age, preoperative KPS, tumor size, Ki-67 indexes, and MGMT promoter methylation. The concentration of CD8^+^ TILs was significantly lower in mGBMs (4.82 ± 3.12/HPF, median 4.70/HPF, interquartile range 2.38–6.12/HPF) than in uGBMs (8.11 ± 4.82/HPF, median 7.80/HPF, interquartile range 5.33–11.70/HPF; P=0.002; **Figures 1B, C**).

**Table 2:** Comparison of mGBMs and uGBMs.

Clinical features	mGBMs			uGBMs			*P* value
	No.		%	No.		%	
**Sex**							0.908
Male	45		78.9	24		80	
Female	12		21.1	6		20	
**Age (years)**							0.425
Mean		55.3			53.7		
SD		8.9			8.1		
**Pre-op KPS**							0.745
≤ 80	36		63.2	20		66.7	
> 80	21		36.8	10		33.3	
**Tumor size (cm^3^)**							0.394
Mean		21.8			19.4		
SD		13.3			11.7		
**Ki-67**							0.975
< 50%	34		59.6	18		60.0	
> 50%	23		40.4	12		40.0	
**MGMT promoter**							0.206
Methylated	29		50.9	11		36.7	
Unmethylated	28		49.1	19		63.3	
**CD8^+^ TILs (/HPF)**							
Mean	4.82	4.82			8.11		*0.002*
SD		3.12			4.82	

MGMT, O(6)-methylguanine-DNA-methyltransferase; mGBM, multifocal and multicentric glioblastoma; pre-op KPS, preoperative Karnofsky performance status; TILs, tumor-infiltrating lymphocytes; uGBM, unifocal GBM.

In mGBMs, no significant difference in the concentration of CD8^+^ TILs was observed among foci from the same patient (P>0.150). The presence of CD8^+^ TILs was similar between multifocal and multicentric GBMs (4.60 ± 2.63/HPF *vs.* 4.97 ± 3.07/HPF, P=0.885; **Figures 1D–F**). The levels of CD8^+^ TILs did not vary significantly according to sex, age, tumor size, tumor resection, Ki-67, MGMT promoter methylation, or postoperative radiotherapy or chemotherapy. However, CD8^+^ TIL levels were significantly correlated with preoperative KPS, and low CD8**^+^** lymphocytic infiltration was more frequently found in patients with KPS ≤ 70 (P=0.018; **Table 3**; **Figure 1G**).

**Table 3:** Correlation of CD8^+^ TIL levels and clinical features.

Clinical feature	Low CD8^+^ TILs		High CD8^+^ TILs		P
	No.	%	No.	%	
**Total patients**	28	49.1	29	50.9	
**Sex**					0.103
Male	25	89.3	20	69.0	
Female	3	10.7	9	31.0	
**Age, years**					0.234
Young, ≤55	12	42.9	17	58.6	
Old, >55	16	57.1	12	41.4	
**Pre-op KPS**					*0.018*
Low, ≤70	22	78.6	14	48.3	
High, >70	6	21.4	15	51.7	
**Tumor size, cm^3^**					0.352
Small, ≤22	12	42.9	16	55.2	
Big, >22	16	57.1	13	44.8	
**Tumor resection**					0.055
GTR	14	50.0	17	58.6	
Partial resection	11	39.3	4	13.8	
Biopsy	3	10.7	8	27.6	
**Ki-67**					0.872
≤50%	17	60.7	17	58.6	
>50%	11	39.3	12	41.4	
**MGMT promoter**					0.689
Methylated	15	53.6	14	48.3	
Unmethylated	13	46.4	15	51.8	
**Post-op Radiotherapy**					0.934
With	8	28.6	8	27.6	
Without	20	71.4	21	72.4	
**Post-op Chemotherapy**					0.106
With	14	50.0	21	72.4	
Without	14	50.0	8	27.6	

MGMT, O(6)-methylguanine-DNA-methyltransferase; post-op, postoperative; pre-op KPS, preoperative Karnofsky performance status; TILs, tumor-infiltrating lymphocytes.

### Prognostic Value of CD8^+^ TILs in mGBMs

**Figure 2:**
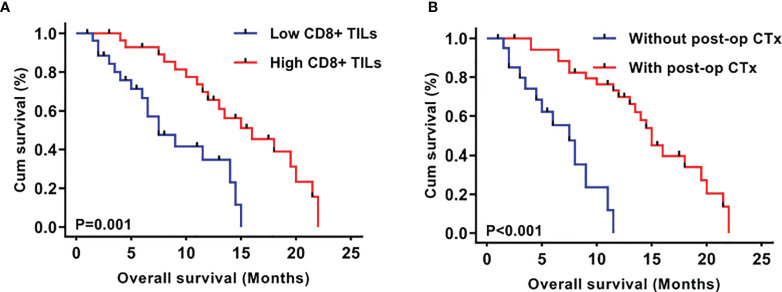
Kaplan–Meier plots of overall survival stratified by CD8^+^ TIL level **(A)** and post-operative chemotherapy **(B)**.

To investigate the prognostic significance of CD8^+^ TILs in this series of mGBMs, the median concentration of CD8^+^ TILs (4.70/HPF) was used as the cutoff value to define a low-infiltration group (n=28) and a high-infiltration group (n=29) for survival analysis. In the Kaplan–Meier analysis, low CD8^+^ TILs were significantly associated with shorter overall survival (OS, median OS 6.3 months *vs.* 12.5 months, P=0.001; **Figure 2A**). Meanwhile, postoperative chemotherapy was significantly associated with prolonged OS (median 9.5 months *vs.* 8.5 months, P<0.001; **Figure 2B**).

As shown in **Table 4**, in univariate analyses, the *P* values of postoperative chemotherapy, CD8^+^ TILs, MGMT promotor methylation, and age were less than 0.2 and were consequently included into multivariate analyses. Multivariate Cox analyses demonstrated that CD8^+^ TILs (P=0.0004, HR 4.404, 95% CI 1.954-9.926) and postoperative chemotherapy (P=0.0002, HR 6.076, 95% CI 2.330-15.842) were independent prognostic factors in mGBMs. Consistent with previous reports (7, 10), no significant difference was observed in the OS between patients with multifocal and multicentric GBMs (P=0.462).

**Table 4:** Univariate and multivariate analyses of different prognostic parameters for overall survival of 57 mGBM patients.

	Univariate survival analysis			Multivariate survival analysis		
	P	HR	95% CI	P	HR	95% CI
Age	0.123	0.577	0.287-1.161	0.225	0.636	0.306-1.321
Pre-operative KPS	0.396	0.736	0.363-1.493			
Tumor size	0.258	1.480	0.750-2.918			
Tumor resection	0.339	-	-			
Ki-67	0.468	0.771	0.382-1.556			
MGMT promoter methylation	0.120	1.754	0.864-3.559	0.150	1.737	0.819-3.681
Type of mGBM	0.462	1.353	0.605-3.026			
CD8^+^ TILs	*0.001*	*3.671*	*1.679-8.026*	*0.0004*	*4.404*	*1.954-9.926*
Post-operative radiotherapy	0.236	1.605	0.734-3.510			
Post-operative chemotherapy	*0.0002*	*7.012*	*2.820-17.438*	*0.0002*	*6.076*	*2.330-15.842*

mGBM, multifocal and multicentric glioblastomas; MGMT, O(6)-methylguanine-DNA-methyltransferase; KPS, preoperative Karnofsky performance status; TILs, tumor-infiltrating lymphocytes.

## Discussion

Although GBMs have been extensively investigated, few studies have focused on multifocal and multicentric GBMs (mGBMs) possibly due to their rare occurrences. Since the majority of newly diagnosed GBMs are unifocal, mGBMs may have distinct molecular mechanisms, microenvironmental characteristics, and clinical courses (10, 11), which have not been clearly elucidated. Moreover, current standard radio-chemotherapy shows inferior therapeutic effects in mGBMs compared with those in uGBMs (15). Therefore, the development of novel mGBM-targeted treatment strategies is clinically relevant.

Previous studies have demonstrated that the immunosuppressive TME plays an important role in the development and progression of GBMs (24, 25). A hallmark of GBM-induced immunosuppression is the inhibition of CD8^+^ effector T-cell mediated anti-tumor responses (26, 27). A number of GBM-derived factors trigger reprogramming of immune cells and inhibit the accumulation and activation of cytotoxic CD8^+^ TILs (24, 28). Although some studies have failed to show a relation between CD8^+^ TILs and clinical outcomes (20, 21), several studies have observed a significant correlation between decreased CD8^+^ TILs and poorer patient survival in GBMs (22, 23, 29). In addition, previous studies did not investigate mGBMs and uGBMs separately. Therefore, in the present study, we specifically examined the CD8^+^ TILs in mGBMs. We found that mGBMs were associated with decreased CD8^+^ lymphocytic infiltration compared with uGBMs. Moreover, lower CD8^+^ TIL levels predicted shorter survival times in mGBM patients. Decreased CD8^+^ TIL levels indicate a more serious local immunosuppressive TME, which may facilitate the development of multifocality and promote the immune evasion of tumor cells. Therefore, inhibition of CD8^+^ lymphocytic infiltration might provide an immuno-microenvironmental basis for the formation and progression of multiple lesions of mGBMs. Moreover, our present results showed that CD8^+^ TIL levels were associated with preoperative KPS in mGBM patients, which suggests an interaction of systemic status and the local immune TME. Nevertheless, further studies are required to better understand the intrinsic mechanisms of this immunosuppression. Our present results also indicate that the immunomodulatory mechanism employed by mGBMs poses a considerable challenge to immunotherapy, since effective immunotherapy often requires the participation of functionally active CD8^+^ T cells (28, 30). Thus, counteracting the impairment of CD8^+^ TILs and promoting a sustained tumor-cell-directed cytotoxic T-cell response are necessary to overcome such immunosuppression and to establish efficacious immunotherapeutic treatments in mGBM patients.

Currently, specific clinical guidelines for the standard treatment of mGBMs are still unavailable (1). Unfortunately, mGBMs are treated in the same way as uGBMs, although they are biologically and clinically different from each other (5, 10, 11). Therefore, the efficacies of surgical treatment and radio-chemotherapy should be re-evaluated in mGBM patients. For example, the role of surgery for uGBMs has been well demonstrated by previous studies, and GTR significantly improves OS (31, 32). However, the optimal surgical management of mGBMs remains controversial (5). Some authors support stereotactic biopsy as the first choice, showing that radical resection increases postoperative morbidity without survival benefits (33–35); while other authors propose aggressive resection, reporting that cytoreductive surgery strongly influences OS in mGBM patients (6, 36, 37). In addition, the prognostic value of radiation therapy has not been clarified in mGBM patients, and the efficacies of whole-brain radiotherapy and 3D conformal radiotherapy are still under debate (13, 38, 39). In the present study, we found that TMZ chemotherapy was associated with improved OS of patients with mGBMs, which is consistent with findings from previous reports (15, 38). Therefore, TMZ systemic therapy is effective and beneficial and should be a major component of forthcoming therapeutic strategies for mGBMs.

### Limitations

The present study had some limitations: This was a retrospective study, and the number of included cases was relatively small. Whenever possible, prospective studies with large sample sizes should be performed to draw stronger conclusions. Given that mGBMs rarely occur, accumulation of such cases is clinically relevant and important. Moreover, the quantification of CD8^+^ TILs is difficult and our approach may still introduce various kinds of bias, including sampling bias, to the study.

## Conclusions

In this study, we demonstrated decreased CD8^+^ lymphocytic infiltration in mGBMs and potential prognostic significance of CD8^+^ TIL levels in mGBM patients. Our results suggest that the local immunosuppressive TME might affect the development and progression of mGBMs.

## Data Availability Statement 

The raw data supporting the conclusions of this article will be made available by the authors, without undue reservation.

## Ethics Statement

This study was approved by the institutional review board of the First Hospital of China Medical University (AF-SOP-07-1.1-01).

## Authors Contributions 

RW, YS, TH, XW, and LK cooperated to complete the experiment. RW, TH, YJ, DZ, JY, and SH contributed to the collection and analysis of data. RW, YS, TH, XW, and YJ participated in drafting the text and figures. SH and LK designed the study and gave indispensable guidance in drafting the manuscript. All authors contributed to the article and approved the submitted version.

## Funding

This work was supported by the Liaoning Revitalization Talents Program (no. XLYC1807253) and the National Natural Science Foundation of China (nos. 81772653 and 81402045).

## Conflict of Interest

The authors declare that the research was conducted in the absence of any commercial or financial relationships that could be construed as a potential conflict of interest.

## Publisher’s Note

All claims expressed in this article are solely those of the authors and do not necessarily represent those of their affiliated organizations, or those of the publisher, the editors and the reviewers. Any product that may be evaluated in this article, or claim that may be made by its manufacturer, is not guaranteed or endorsed by the publisher.
